# Total antioxidant level is correlated with intra-ocular pressure in patients with primary angle closure glaucoma

**DOI:** 10.1186/1756-0500-7-163

**Published:** 2014-03-19

**Authors:** Khaled K Abu-Amero, Taif Anwar Azad, Ahmed Mousa, Essam A Osman, Tahira Sultan, Saleh A Al-Obeidan

**Affiliations:** 1 Department of Ophthalmology, College of Medicine, King Saud University, PO Box 245, Riyadh 11411, Saudi Arabia; 2 Department of Ophthalmology, College of Medicine, Jacksonville, FL, USA

**Keywords:** PACG, Oxidative stress, Anti-oxidant, Glaucoma, Saudi Arabia

## Abstract

**Background:**

To evaluate total antioxidant status (TAS) in the plasma of primary angle closure glaucoma (PACG) patients and to compare it to that of the control group. Additionally, we aim to investigate the association of various PACG clinical indices with TAS level.

**Methods:**

Plasma samples were obtained from 139 PACG patients and 149 glaucoma-free controls of matching age, sex, and ethnicity. TAS in all samples was determined by spectrophotometric and enzyme-linked immunosorbent assay methods. We studied the possible association of the TAS level with various clinical indices relevant to PACG.

**Results:**

The mean (±SD) total antioxidant (TAS) value was almost similar in patients 1 (±0.22) compared to controls 0.97 (±0.43); *p* = 0.345. Among cases, mean TAS concentration showed a statistically significant lower pattern among subjects with glaucoma onset at the age of ≤ 50 years (*p* = 0.037) and female subjects (*p* = 0.014) as well as having a family history of glaucoma (*p* = 0.010). Interestingly, a statistically significant inverse correlation was detected between TAS concentration and intra ocular pressure (IOP), (R = -0.14, *p* = 0.037).

**Conclusions:**

The inverse correlation of TAS level with IOP, highlights TAS potential role as a predictive-marker for PACG-severity.

## Background

Although glaucoma embodies a heterogeneous group of optic neuropathies, all types are defined by progressive and irreversible degeneration of the optic nerve with gradual visual field loss. Within this group, primary angle-closure glaucoma (PACG) is a type of glaucoma characterized by a narrow iridocorneal angle resulting in a blockage of the aqueous outflow structures. It seems that there is an anatomical and physiological predisposition to PACG. Thus, shallow anterior chamber depth is considered an anatomically inheritable risk factor for this illness [1]. Congruently, Eastern Asian ascendance, hyperopia and female gender significantly predispose to this disease [2]. It has been estimated that in Saudi Arabia, 40% of glaucoma patients belong to the PACG type [3]. This percentage is closer to those calculated for Asian than for European populations [4]. Although a hereditary component for PACG exists, causative genes have not yet been identified until recently where a significant association at three new loci: rs11024102 in *PLEKHA7* (per-allele odds ratio (OR) = 1.22; P = 5.33 × 10(-12)), rs3753841 in *COL11A1* (per-allele OR = 1.20; P = 9.22 × 10(-10)) and rs1015213 located between *PCMTD1* and ST18 on chromosome 8q (per-allele OR = 1.50; P = 3.29 × 10(-9) were reported [5]. The three SNPs may explain in part some aspects of the PACG pathogenesis, but not all. Oxidative stress has been implicated to cause increased IOP by triggering TM degeneration and thus contributing to alterations in the aqueous outflow pathway. Indeed, treatment with hydrogen peroxide (H_2_O_2_) impairs TM cell adhesion to the extracellular matrix and causes rearrangement of cytoskeletal structures [6]. In humans, in vivo experiments demonstrated that oxidative DNA damage is significantly more abundant in the TM cells of glaucoma patients. Additionally, both increased IOP and visual field damage were significantly related to the amount of oxidative DNA damage affecting TM cells [6,7]. The antioxidant status of biologic samples is regarded as an indicator of oxidative stress, and the measurement of total antioxidant status (TAS) is one of the most commonly used and useful procedures to test for prediction of oxidative status [8]. Here, we investigated the total antioxidant status as a possible contributor and potential marker for PACG.

## Methods

### Study population

The study adheres to the tenets of the Declaration of Helsinki, and all participants signed an informed consent. The study was approved by the College of Medicine Ethical Committee (approval number # 08-657). Saudi Arab participants with clinically diagnosed PACG and healthy controls were recruited into the study at King Abdulaziz University Hospital (KAUH) in Riyadh, Saudi Arabia.

We recruited 139 Saudi PACG patients (cases) who satisfied strict clinical criteria for PACG which includes the following: at least three of the following: 1) clinical documentation of angle closure, defined as the presence of appositional or synnechial closure of the anterior chamber angle involving at least 270 degrees by gonioscopy in either eye; 2) intraocular pressure elevated to a level ≥21 mmHg measured by Goldmann applanation tonometry; 3) evidence of characteristic glaucomatous optic disk damage with excavation of the disc causing a cup-to-disk ratio (c/d) vertically of at least 0.70 in at least one eye; and 4) characteristic peripheral visual field loss including nerve fiber bundle defects (nasal step, arcuate scotoma, paracentral scotoma) or advanced visual field loss (central and/or temporal island of vision) as tested by Humphrey Field Analyzer in those patients with vision better than 20/200 or Goldmann Manual Perimetry in those with worse vision.

Exclusion criteria included: 1) secondary angle closure glaucoma; 2) presence of pseudoexfoliation syndrome even if coexistent with angle closure; 3) another cause of optic nerve injury affecting either eye; 4) significant visual loss in both eyes not associated with glaucoma; 5) inability to visualize the fundus for optic disk assessment; or 6) refusal to participate.

Patients were recruited from the glaucoma clinic at King Abdulaziz University Hospital (KAUH) after signing an informed consent approved by the institutional review board (proposal number # 08-657).

A second group (n = 149) of healthy Saudi Arabs controls (Controls group) free from glaucoma by examination were recruited. Entry criteria for those subjects were age >40, normal IOP, open angles on gonioscopy, and normal optic nerves on examination.

### Plasma preparation and storage

Blood samples were collected in EDTA (ethylenediaminetetraacetic acid) tubes. The tubes were centrifuged at 5500 *x*g for 5 min. The plasma layer was separated and stored at -80°C until use and the Buffy layer was used for DNA extraction.

### Plasma total antioxidant status

A widely used colorimetric-based assay available from Randox (Randox Laboratories Ltd, UK) was used to evaluate the plasma total antioxidant status. The assay involves brief incubation of ABTS® (2,2′-Azinobis-di [3-ethylbenzthiazoline sulphonate]) with peroxidase (metmyoglobin) and hydrogen peroxide, resulting in the generation of ABTS®^+^ radical cations. The method detects a reduction in the generation of the ABTS®^+^ radical cations by plasma antioxidants, and the decrease in the generation of ABTS®^+^ radical cations is proportional to their total antioxidant concentration. The assay was performed in duplicate on an automated biochemical analyzer, ChemWell-T (Awareness Technology Inc., FL, USA), as per the manufacturer’s instructions (Randox Laboratories, UK). The analyzer was programmed using a ChemWell-T Assay Editor in the standard assay mode to add 200 μl of chromogen (metmyoglobin and ABTS®) and 4 μl of plasma sample/standard control/distilled water, incubate at 37°C for 10s, and read at 630 nm. This was followed by the addition of 40 μl of substrate (hydrogen peroxide in stabilized form), incubation at 37°C for exactly 3 min, and measuring absorbance at 630 nm. A standard control (6-hydroxy-2,5,7,8-tetramethylchroman-2carboxylic acid) provided in the kit (Cat. No. NX 2332: lot specific concentration = 2.08 mmol/L) was used for calibration. The total antioxidant status (TAS) was expressed as mmol/L.

### Statistical analysis

Statistical analysis was performed using SPSS version 19.0 (IBM Corp., Armonk, New York, USA). Descriptive analysis was conducted to describe the distribution of the sample by major clinical and demographic study indices. Shapiro test was applied to investigate whether continuous variables were normally distributed. Pearson correlation coefficient was calculated to investigate potential correlations between the TAS value and different clinical and demographic indices (Spearman test when indicated). Student’s T test was used to compare the mean TAS between cases and control across groups, while Mann Whitney U test was used to compare the mean TAS within groups (between different categories of severity). A *p*-value of <0.05 was considered of statistical significance, while all tests were supported by the corresponding 95% confidence intervals.

## Results

In the current study, we recruited a total number of 288 subjects in the age interval of 40 years and older; where 139 subjects were of confirmed clinical diagnosis as having PACG and were considered as cases, while other exactly matching 149 subjects were diagnosed as glaucoma free and were categorized in the current study as controls. Among the cases, 85 (61.2%) were males and 54 (38.8%) were females, while among controls; 91 (61.1%) were males and 58 (38.9%) were females. Comparing these two gender ratios, there were not statistically different (*p* = 0.988). The mean (±SD) age of cases was 63.1 (±9.4), median; 64, range [40 – 87] while the mean (±SD) age of controls was 61.1 (±10.8), median; 60, range [40 – 93]. This minor difference in the mean age (2 years) was not also statistically significant (*p* = 0.091).

The mean (±SD) total antioxidant (TAS) level was slightly higher among cases; 1 (±0.22) than controls; 0.97 (±0.43) with a mean difference of 0.038 (95% CI: - 0.041 – 0.116] however, insignificantly (*p* = 0.345).

Moreover, in terms of glaucoma specific indices at the time of presentation; family history of glaucoma was founded in 12/139 (8.6%), while consanguinity was common among 6/139 (4.3%) of the total recruited subjects while 7 (5%) cases were unilateral and 132 (95%) were bilateral. The mean (±SD) age at first onset of the disease was 54.7 (±19.1), mean intraocular pressure (IOP) was 18.2 (±7.5); where 74/139 (53.2%) subjects are under antiglaucoma mediation, mean vertical cup/disc ratio (CDR); 0.68 (±0.23), mean number of antiglaucoma medications; 1.9 (±1.6), and LogMAR visual acuity; 0.65 (±0.81) (Table 1).

**Table 1:** Cases and controls, distributed per demographic and clinical indices at presentation

**Index**	**Category**	**Case**	**Control**	**P value**
		**No. (%)**	**No. (%)**	
Gender	Male	85 (61.2%)	91 (61.1%)	0.988
	Female	54 (38.8%)	58 (38.9%)	
		**Mean (SD)**	**Mean (SD)**	**P value**
Age		63.1 (9.4)	61.1 (10.8)	0.091
TAS		1 (0.22)	0.97 (0.43)	0.345
Age at Onset		54.7 (19.1)	N/A	-
IOP		18.2 (7.5)	N/A	-
CDR		0.68 (0.23)	N/A	-
No. of Mediations		1.9 (1.6)	N/A	-
LogMAR VA		0.65 (0.81)	N/A	-

TAS unit of measurement is mmol/L.

Meanwhile, assessment of other systemic clinical indices at presentation showed that there were a comorbidity of such diseases among cases as follows: 37/139 (26.6%) were diabetic, 35/139 (25.2%) were having hypertension, while hypercholesterolemia was found among 2 (1.4%) cases, coronary artery diseases in one (0.7%) case, only two cases were smokers, in addition to 4 other miscellaneous cases. Seventeen subjects (12.2%) were aware of having glaucoma, with a mean (±SD) awareness duration of 47.1 (±42.9) months. Among the glaucoma free group (controls): 51/149 (34.2%) were diabetic, and 54/149 (36.2%) were suffering hypertension. There was no statistically significant difference between cases and controls neither in the prevalence of diabetes mellitus nor in the prevalence of hypertension (*p* = 0.204 and 0.059 espectively).

Table 2, shows the distribution of mean TAS level per different categories of demographic and clinical characteristics. Among cases, mean TAS concentration showed a statistically significant lower pattern among subjects with glaucoma onset at the age of ≤ 50 years (*p* = 0.037) and female subjects (*p* = 0.014) as well as having a family history of glaucoma (*p* = 0.010). Nonetheless, there was no association with age groups, laterality, consanguinity, IOP, CDR, vision and numbness of antiglaucoma medication in addition to all the other systemic characteristics (Table 2).

**Table 2:** Distribution of mean TAS level per different categories of demographic and clinical indices

**Index**	**Category**	**Case**	**P value**
		**Mean (SD)**	
Age (years)	≤50	0.96 (0.25)	0.297
	>50	1.1 (0.22)	
Gender	Male	1.04 (0.2)	0.014
	Female	0.95 (0.23)	
Age at Onset (years)	≤50	1.33 (0.17)	0.037
	>50	1.06 (0.22)	
Laterality	Unilateral	0.98 (0.23)	0.728
	Bilateral	1.01 (0.22)	
FH of Glaucoma	No	1.02 (0.22)	0.010
	Yes	0.89 (0.19)	
Consanguinity	No	1.01 (0.22)	0.106
	Yes	0.91 (0.24)	
Diabetes	No	1.0 (0.22)	0.949
	Yes	1.0 (0.22)	
Hypertension	No	0.99 (0.21)	0.258
	Yes	1.03 (0.23)	
IOP	≤ 21	1.02 (0.21)	0.350
	> 21	0.99 (0.12)	
CDR	≤ 0.65	1.03 (0.21)	0.338
	> 0.65	0.99 (0.22)	
No. of Medications	≤ 2	1.01 (0.23)	0.231
	> 2	1.06 (0.17)	
LogMAR Vision	≤ 20/60 (0.48)	0.99 (0.22)	0.136
	> 20/60 (0.48)	1.04 (0.23)	

TAS unit of measurement is mmol/L.

**Figure 1:**
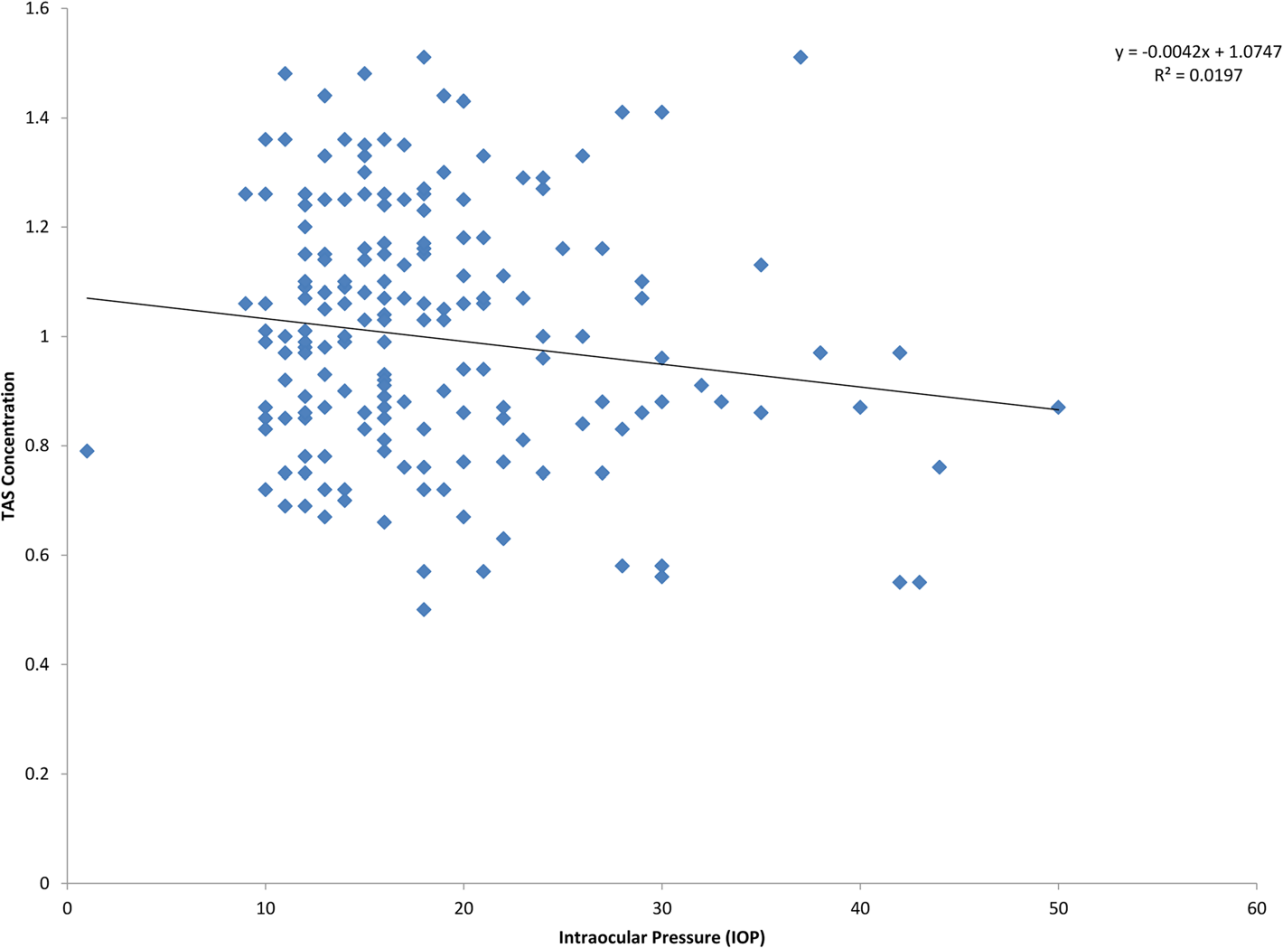
Correlation between IOP and TAS concentration.

Investigating the potential correlation between TAS concentration and the glaucoma indices, a statistically significant inverse correlation was detected between TAS concentration and IOP (R = -0.14, *p* = 0.037) (Figure 1). Meanwhile, TAS concentration was not significantly correlated to vertical CDR (R = -0.013, p = 0.851), while the correlation test was not applicable to other variables due to being not normally distributed.

## Discussion and conclusions

We obtained plasma samples from 139 extensively diagnosed PACG patients and 149 controls. Our control group was carefully selected in terms of age, sex, ethnicity, smoking status and other diseases (diabetes, hypertension and hypercholesterolemia) which could influence TAS levels. The two groups were matching for those factors and this was of particular importance, as those factors can influence the TAS level, and thus can affect the results independent of the glaucoma status [9].

We then proceeded with measurement of TAS levels in both groups. Although TAS level was higher in the Patient group compared to the controls, this difference was not statistically significant. This was in contrast to our previous results where TAS level was decreased in a group of patients with psueduoexfoliation glaucoma (PEG) [10]. This may indicate that oxidative stress represented indirectly by the TAS levels, play a bigger role in case of PEG than for PACG. Oxidative stress which is linked strongly to mitochondrial abnormalities (such as decreased mitochondrial respiration, mtDNA mutations and mtDNA copy number alterations) were demonstrated in POAG and PEG cases but not in PACG, thus enforcing our current observation that oxidative stress may play a minor role in PACG development compared to other types of glaucoma. It is also possible that TAS decreased levels are more linked to particular clinical indices important for PACG rather than for the disease as a whole. Optic nerve injury in PACG has been attributed primarily to elevated IOP caused by anatomic changes in the anterior [11] and posterior [12] globe, in contrast with the molecular and biochemical abnormalities suspected in POAG [13]. Perhaps for this reason, the role of oxidative stress in PACG has received limited attention demonstrated by the fact that this is the first study to investigate the TAS level in PACG patients. This is despite reports that PACG may be present in 3.9 million people around the world [14].

When we investigated the potential correlation between TAS and glaucoma indices, we found a statistically significant inverse correlation between TAS and IOP. This is interesting, since optic nerve injury in PACG is attributed primarily to elevated IOP.

The literature shows inconsistent findings regarding antioxidant activity in serum and aqueous humor in glaucoma patients. Yildrim and colleagues studied 40 patients with glaucoma and found no association between glaucoma and systemic myeloperoxidase or catalase enzyme activity [15]. Yuki and colleagues found an increase in the serum total antioxidant status of patients with normal-tension glaucoma compared to matching controls [16]. In contrast, Sorkhabi et al. showed that the serum level of TAS in patients with primary open angle glaucoma was lower than that of cataract controls [17]. Gherghel and colleagues [18] concluded that glaucoma patients exhibit low levels of circulating glutathione, suggesting compromised oxidative defense. The only study of total reactive antioxidant potential (TRAP) and antioxidant enzymes in aqueous humor was performed by Ferreira and colleagues, and showed significantly decreased TRAP values and increased superoxide dismutase and glutathione peroxidase activity in glaucoma patients [8]. The antioxidant defense system comprises a variety of molecules: enzymes such as superoxide dismutase, catalase, or glutathione peroxidase, that are capable of catalytically removing free radicals and other reactive species; proteins, such as transferrins or haptoglobins, that minimize the availability of pro-oxidants such as iron or copper ions; heat shock proteins that protect biomolecules against damage; and low-molecular mass molecules such as α-tocopherol, ascorbic acid, or glutathione capable of scavenging ROS and RNS. The composition of antioxidant defenses differs from tissue to tissue and from cell type to cell type [19]. All of these compounds and more exist in human plasma. Antioxidants that can be found in human plasma vary, and can be summarized mainly in the following compounds: albumin, ceruloplasmin, ferritin, ascorbic acid, α-tocopherol, β-carotene, lycopene, reduced glutathione, bilirubin, glutathione peroxidase, uric acid, catalase, and superoxide dismutase [20]. The exact mechanism of how oxidative stress contributes to glaucoma pathogenesis remains speculative. Glaucomatous optic neuropathy implies loss of retinal ganglion cells, including their axons, and a major tissue remodeling, especially in the optic nerve head. Although increased intraocular pressure is a major risk factor for glaucomatous optic neuropathy, there is little doubt that other factors such as ocular blood flow play a role as well [21]. Mechanisms leading to glaucomatous optic neuropathy are not yet clearly understood. There is, however, increasing evidence that both an activation of glial cells and oxidative stress in the axons may play an important role [22]. Glial cells may be activated by mechanical stress via activation of the epidermal growth-factor receptor, or by ischemic stress via an increase in endothelin.

We have to acknowledge the following limitations in our study. First, our total antioxidants method employed here measured the total antioxidant status and not a particular compound or byproduct. A detailed examination of those individual antioxidants separately might help to identify a particular antioxidant that is severely decreased and thus provide a new therapeutic agent for glaucoma. Second, the systemic decrease in antioxidants might not reflect the exact situation at the anterior segment structures, which are exposed to free radicals and thus more directly involved in the formation and development of glaucoma through the oxidative stress mechanism.

Since measurement of TAS levels in the plasma is usually straightforward and relatively not expensive, TAS level can be used routinely as a marker for PACG especially if our results are confirmed in different ethnicities and larger cohorts.

## Abbreviations

c/d: Cup-to-disk ratio; EDTA: Ethylenediaminetetraacetic acid; H2O2: Hydrogen peroxide; IOP: Intra ocular pressure; KAUH: King Abdulaziz University Hospital; OR: Odds ratio; PACG: Primary angle closure glaucoma; POAG: Primary open angle glaucoma; TAS: Total antioxidant status; TRAP: Total reactive antioxidant potential.

## Competing interests

The authors declare that they have no competing interests.

## Authors’ contributions

KKA Conception and design, or analysis and interpretation of data, drafting the article or revising it critically for important intellectual content and final approval of the version to be published; TAA Analysis and interpretation of data; AM Conception and design, or analysis and interpretation of data, drafting the article or revising it critically for important intellectual content and final approval of the version to be published; EAO conception and design, or analysis and interpretation of data; TS Analysis and interpretation of data; SAA Drafting the article or revising it critically for important intellectual content and final approval of the version to be published. All authors read and approved the final manuscript.
